# Safety and benefits of adult-worn slings and baby carriers: a narrative systematic review to inform guidance for parents

**DOI:** 10.1136/bmjpo-2026-004693

**Published:** 2026-06-03

**Authors:** Sophie R. Lovell-Kennedy, Rebecca L J Strong, Laura M Grieve, Helen L Ball

**Affiliations:** 1 Durham Infancy & Sleep Centre, Anthropology, Durham University, Durham, UK

**Keywords:** Infant, Mortality, Mothers, Caregivers

## Abstract

**Background:**

Adult-worn slings and carriers (AWSC) are common components of infant care globally. Several widely publicised deaths of babies have occurred during AWSC-use, raising concerns about their safety and the strength of existing parental guidance. As part of a wider project to inform recommendations for UK guidance for the safe use of AWSC, this review was undertaken to assess the risks, benefits, harms and parental motivations for AWSC-use.

**Methods:**

A systematic review of databases PubMed, Web of Science and CINAHL (2000–2024) was undertaken following Preferred Reporting Items for Systematic Reviews and Meta-Analyses (PRISMA) guidelines. Searching and sifting were conducted between January and April 2025, followed by a narrative summary and analysis of extracted data.

**Results:**

62 papers were included in the final review. Most research involved small numbers of participants and was undertaken in North America but papers were included from Europe, Africa, Asia and Australia. The reviewed studies found benefits associated with AWSC-use for both caregivers and infants. However, AWSC were also linked to infrequent accidental infant deaths (primarily asphyxia) and injuries (namely falls) co-occurring with AWSC being poorly fitting or positioned. Ill-fitting AWSC were associated with pain and discomfort for adult wearers. Parents’ primary motivation for AWSC-use was the desire to be responsive to their baby while enabling their own mobility.

**Conclusions:**

The research reviewed confirms that multiple benefits accrue to babies and caregivers with AWSC-use. There is also evidence of infrequent accidental infant deaths and injury linked to poor fit and improper use of AWSC. Parents need clear guidance on how to babywear safely while achieving their goals of responsiveness and mobility. National guidance focusing on infant safety, specifically highlighting the need for appropriate use and fit of AWSC to prevent asphyxia and falls, should be provided to all parents and should signpost them to specialist AWSC education and support.

**PROSPERO registration number:**

CRD420251001795.

**WHAT IS ALREADY KNOWN ON THIS TOPIC**

**WHAT THIS STUDY ADDS**

- Accidental asphyxiation of young babies is the key risk for infant deaths, and falls are the main risk for infant injuries during AWSC-use.
- No peer-reviewed research has examined when and why parents choose to use AWSC, or how they access safety information and support.

**HOW THIS STUDY MIGHT AFFECT RESEARCH, PRACTICE OR POLICY**

- This systematic review evidences the need for research on parent knowledge and practice around AWSC-use.
- It provides evidence to inform parental information campaigns to improve AWSC safety awareness and signpost to appropriate sources of support.

## Introduction

 Adult-worn slings and baby carriers (AWSC) have been used across cultures and history.^1^,^5^ Human infants experience prolonged helplessness, requiring close contact with caregivers to meet their biological needs. Carrying enables this contact, just as our primate relatives cling to their mothers’ fur.^6^ Many babies today spend substantial time held in AWSC in contact with a caregiver,^7 8^ but infant-carrying methods vary cross-culturally.^9^

Recently, AWSC-use (or ‘babywearing’) has grown in popularity across the Global North^10^ as responsive parenting practices have become widespread.^11 12^ Global market reports suggest that the AWSC industry is growing at 5%–10% per annum.^13^ However, several widely publicised deaths of babies while in AWSC or following injuries incurred while in AWSC^14^,^16^ have raised concerns about AWSC safety and resulted in calls to strengthen guidance.^17^

At the outset of this project, no evidence-based national UK guidance on AWSC safety existed; only the TICKS guidance (created by the UK Sling Consortium) was available, but this was not comprehensive and lacked underpinning evidence. The NHS website directed parents to TICKS, to the Royal Society for the Prevention of Accidents and to The Lullaby Trust.^18^ A nationwide network of sling libraries provided support for AWSC-use but was not well-known or used. It was unclear whether independent guidance provided by AWSC manufacturers, retailers, babywearing consultants, sling libraries and charities concerned with infant safety was comprehensive, consistent, accessible and used by families.

As part of a wider project to inform recommendations for UK national guidance, this narrative systematic review was undertaken to assess the findings of international peer-reviewed publications from 2000 to 2024 into AWSC-use, including risks, benefits, harms and parental motivations.

## Methods

Academic literature on parental and infant outcomes regarding AWSC-use was systematically accessed and reviewed following PRISMA (Preferred Reporting Items for Systematic Reviews and Meta-Analyses) 2020 guidelines and reporting checklist.^19 20^ Three databases providing wide coverage of worldwide health-related and clinical literature (PubMed, Web of Science and CINAHL) were searched for terms shown in table 1 using truncation, wildcards and Boolean logic. Citation mining was used to identify further literature. Titles of peer-reviewed articles published between January 2000 and December 2024 were retrieved and imported into Covidence software for screening and data extraction.

**Table 1:** Search terms

Key search terms	Database	Search string
Bab* Infan* Sling* Wear* Wrap* Carrier	PubMed (April 2025)	Search strategy: (((baby[Title/Abstract] OR babies[Title/Abstract] OR infan*[Title/Abstract)) AND (sling*[Title/Abstract] OR wear*[Title/Abstract] OR wrap*[Title/Abstract])) OR (baby ads2 wearing[Title/Abstract] OR baby ads2 sling[Title/Abstract] OR baby ads2 carrier[Title/Abstract] OR baby ads2 wrap[Title/Abstract])) OR (infan* ads2 wearing[Title/Abstract] OR infan* ads2sling[Title/Abstract] OR infan* ads2 carrier[Title/Abstract] OR infan* ads2 wrap[Title/Abstract])
	Web of Science (April 2025)	Search Strategy: (bab* OR infan*) AND (sling* OR wear* OR wrap*) OR (bab* ads2 wearing OR bab* ads2 sling OR bab* ads2 carr* OR bab* ads2 wrap) OR (infan* ads2 wearing OR infan* ads2 sling OR infan* ads2 carr* OR infan* ads2 wrap)
	CINAHL (April 2025)	(bab* OR infan*) AND (sling* OR wear* OR wrap*) OR (bab* ads2 wearing OR bab* ads2 sling OR bab* ads2 carrier OR bab* ads2 wrap) OR (infan* ads2 wearing OR infan* ads2 sling OR infan* ads2 carrier OR infan* ads2 wrap)

After removal of duplicates, abstracts were screened by two researchers (RLJS and SRL-K); those not meeting the inclusion criteria were excluded from further review (table 2). The same researchers then undertook a full-text screening. Any conflicts were resolved by a third researcher (HLB). PhD thesis research was included unless the dataset had also been used in a peer-reviewed article, in which case the article was used instead.

**Table 2:** Inclusion and exclusion criteria

Inclusion criteria (full text screen)	Exclusion criteria	
	Title screening	Abstract screening
Risks or hazards of using slings, wraps or carriers	No mention of infant physical or mental health concerns or issues	No mention of risks or hazards of using slings, wraps or carriers
Parent motivation or perceptions of using slings, wraps or carriers in infant caregiving	No mention of specific caregiving attributes for sling/carrier/wrap use (eg, sleep, feeding, bonding)	No mention of parental motivation or perceptions
Relationship of use of slings/wraps/carriers to infant health and/or SUDI/SUID/SIDS	No mention of infant health or related terms (eg, SUID, SUDI, SIDS, injury, specific illnesses or health concerns)	No mention of slings, carriers or wraps in baby wearing practices in relation to infant health and/or SUDI/SUID/SIDS
Relationship of use of slings/wraps/carriers to parental physical and mental health	No mention of parental physical or mental health concerns or issues	No mention of sling, wrap or carrier practices
Sling, wrap or carrier practices within different countries and/or cultures		Not published in English

SIDS, Sudden Infant Death Syndrome; SUDI, Sudden Unexpected Death in Infancy; SUID, Sudden Unexpected Infant Death.

Data extraction from included papers was completed independently using a data extraction template (see online supplemental file 1) and then compared to ensure consistency.

## Results

**Figure 1:**
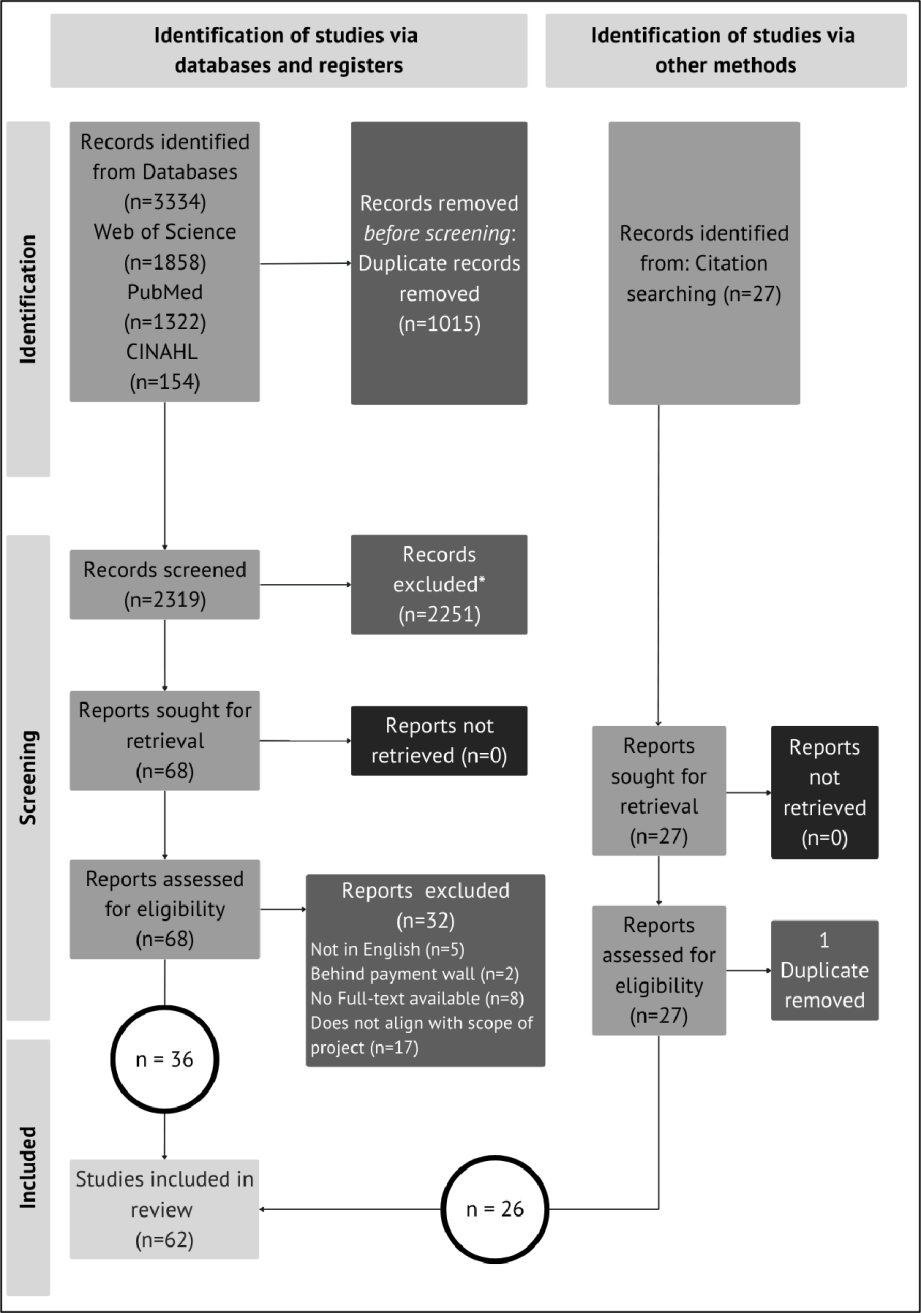
PRISMA (Preferred Reporting Items for Systematic Reviews and Meta-Analyses) flowchart.

Database searching identified 3334 articles; 1015 duplicates were eliminated, 2251 abstracts were screened and excluded with a further 32 excluded following full-text screening, leaving 36 eligible papers. Citation mining identified 26 additional eligible publications. In total, 62 publications were included in the review (figure 1).

The retrieved papers covered a wide array of study types, including experimental studies, death reports, case studies and qualitative studies (see online supplemental files of Summary Table of Included Studies). Given the heterogeneity of the studies and their outcomes, a narrative synthesis was chosen to summarise and analyse the findings. Outcomes from the extracted data clustered into two main themes: (a) benefits, motivations and practicalities of AWSC-use and (b) potential risks of AWSC-use. Within these themes, papers were grouped into six topic areas (table 3) and described narratively. All authors discussed the topics and agreed which papers were included in each grouping.

**Table 3:** Topic and subtopic outcomes identified within themes

	Number of publications (total=62)
Theme 1: Benefits, motivations and practicalities of AWSC-use	39
**Topic 1: Parental beliefs, perceptions and behaviours** Promotion of bonding and attachment Soothing and reduction of crying Facilitating daily activities Enabling infant care for adults with physical disabilities	**14** 8 2 1 3
**Topic 2: Benefits of AWSC-use to infant health and development** Premature or opioid-exposed babies Infection protection Breastfeeding promotion Physical and social development	**21** 9 2 2 8
**Topic 3: Effects on parental mental health**	**4**
Theme 2: Potential risks of using AWSC	23
**Topic 1: Risk of infant death or injury** Asphyxiation Falls	**9** 6 3
**Topic 2: Potential risks to physical infant health**	**2**
**Topic 3: Harm to parental health** Gait and posture of carrying adult Musculoskeletal fatigue and pain in carrying adult Cardiopulmonary and cardiorespiratory systems in carrying adult	**12** 3 6 3

AWSC, adult-worn slings and carriers.

### Benefits, motivations, and practicalities of AWSC-use

Three topics were identified within this theme:

1. Parental beliefs, perceptions and behaviours around AWSC-use.
2. Benefits of AWSC-use to infant health and development.
3. Benefits of AWSC-use to parental mental health

#### Parental beliefs, perceptions and behaviours around AWSC-use

Fourteen articles explored this topic. Parents’ primary motivations for AWSC-use involved facilitating responsive parenting and promoting attachment; soothing distressed infants; facilitating caregiving while undertaking daily activities; and facilitating caregiving for mothers with physical disabilities. Each of the four subtopics is described as follows.

#### Promotion of bonding and attachment

Eight articles explored AWSC-use within attachment- and responsive-parenting contexts, and examined the importance of parent–infant contact for infant socio-emotional development.^1011 21^,^26^ Babies’ biological need for close contact was noted in some papers via indirect evidence such as how AWSC-use helped babies thrive, cry less and breastfeed more.^10 25^ AWSC-use to promote secure attachment and healthy socio-emotional development was directly evidenced in three studies finding: (a) AWSC-use was linked to positive outcomes for bonding, comforting baby, and infant learning^21^; (b) infants of adolescent mothers using AWSC (intervention) were more likely to have secure attachment than infants who were read to (control) and less likely to have disorganised attachment styles^22^; (c) using the Still-Face Paradigm, infants in an AWSC-intervention group spent less time in negative engagement than those in a control group. Comparison of adolescent mothers (n=16) who used AWSC daily for 3 months (intervention group) with a control group (n=17) found that the former were less withdrawn and spent more time in positive engagement and social monitoring with their baby than those in the control group.^23^ All three studies concluded AWSC can promote bonding between infants and adolescent mothers and support secure attachment. One further article suggested AWSC could support healthy socio-emotional development in post-institutionalised children.^24^ Finally, a scoping review found AWSC-use facilitated physical contact, in turn increasing maternal responsiveness.^26^ This was supported by research demonstrating AWSC-use increases maternal responsiveness to infant vocalisations.^11^

#### Soothing and reduction of crying

Two articles found AWSC-use effectively reduces infant crying. Based on observations from clinical practice one peer-reviewed commentary notes that the sensory enrichment AWSC provides reduces excessive infant crying and gastro-oesophageal reflux disease.^27^ This is supported in a comparison of infant crying across three cohorts using a custom-made ‘settle-sling’, a control sling and no sling, which reported the ‘settle sling’ reduced crying.^28^ However, as crying decreased across all groups and those using the ‘settle sling’ had higher baseline crying rates, it is unclear whether the ‘settle-sling’ was more effective in reducing crying than a generic AWSC.

#### Facilitating daily activities

AWSC-use as a tool to facilitate caregiving while undertaking daily activities was explored in one interview study of 24 parents who reported that AWSC-use enabled them to provide infant care while navigating urban environments, in turn, providing freedom and aiding in pregnancy recovery.^29^

#### Enabling infant care for adults with physical disabilities

Three studies gave examples of AWSC-use to facilitate caregiving for mothers with physical disabilities.^30^,^32^ One described how AWSC-use enabled a 34-year-old mother recovering from Guillain-Barré syndrome to independently breastfeed and carry.^30^ Another conducted 22 semi-structured telephone interviews with disabled mothers, detailing how AWSC-use facilitated independent caregiving.^31^ Conversely, an autoethnographic account of disabled motherhood cautioned that AWSC are not designed for individuals with accessibility needs who might require assistance to move their baby in and out of AWSC.^32^

### Benefits of AWSC-use to infant health and development

21 studies examined the effects of AWSC-use on infant health and development, providing evidence that AWSC have positive outcomes for infant physiology and cognitive and language development.

#### Premature or opioid-exposed babies

Kangaroo Mother Care (KMC) involves skin-to-skin (STS) contact between infant and caregiver, with infants positioned naked on caregivers’ chests.^33^ KMC stabilises infant physiology (temperature, respiration, etc) via the caregiver’s body. It is a well-known, effective method for reducing preterm infant mortality from conditions including hypothermia and hypoglycaemia^34 35^; with additional benefits including encouraging breastfeeding and improving sleep.^33^ AWSC are commonly used to support KMC. Nine studies investigated AWSC-use in the context of KMC and STS: four investigated caregiver perceptions and the impact of carrier-type on duration of KMC practice^34^,^37^; three investigated AWSC-use with infants diagnosed with Neonatal Opioid Withdrawal Syndrome (NOWS), also known as Neonatal Abstinence Syndrome^38^,^40^; two investigated AWSC specifically designed to facilitate KMC in hospital and community settings across the USA (four studies), Africa (two studies) and Asia (two studies). Studies also examined acceptability, comfort, ease-of-use and parental fears about infant safety in Global South settings.^34 37^ STS care facilitated through AWSC is an effective non-pharmacological method of managing NOWS symptoms, (eg, infant distress).^38 40^ Two further articles explored new carrier prototypes for use in KMC and STS, seeking to improve health outcomes for premature and low birthweight infants.^41 42^

#### Potential infection protection

Research in Uganda examined the feasibility, acceptability and effectiveness of permethrin-treated traditional wraps (*lesu*) to prevent malaria infection in infants. The treated wraps were considered acceptable and used by all mothers.^43^ A subsequent randomised control trial (RCT) protocol proposed to examine the effect of these wraps on clinical malaria incidence in children.^44^

#### Breastfeeding promotion

Two studies examined the relationship between AWSC-use and breastfeeding outcomes. An Italian RCT assessed whether an educational intervention involving AWSC-use improved breastfeeding duration.^45^ 200 mothers with healthy, term infants received either an AWSC with information and training about its use (intervention), or information about breastfeeding only (control). In the intervention group, 69% of mothers used the carrier daily for at least 1 hour during their infant’s first month of life. Breastfeeding rates were similar in both groups at discharge from hospital, but significantly more mothers in the intervention group breastfed at 2 months (72% vs 51%) and 5 months post partum (48% vs 24%), respectively, confirming that AWSC-use with healthy term neonates is associated with greater breastfeeding duration.^45^ A US survey also found that physical contact with infants, including AWSC-use, predicted increased likelihood of self-reported responsive-feeding, and longer exclusive-breastfeeding duration.^46^

#### Physical and social development

Five studies investigated the relationship of AWSC-use with developmental dysplasia of the hip (DDH).^47^,^51^ A congenital, non-fatal, malformation of infant hip joints^49 51^ which can present in numerous conditions including hip dysplasia, subluxation and dislocation of the hip joint.^50^ These studies found that AWSC provide appropriate hip positioning for healthy hip development^48 50^ with hip position in a soft-structured baby carrier replicating the Pavlik harness, a device used to treat DDH.^49 51^ Furthermore, research in Malawi found low DDH incidence was influenced by the high prevalence of back-carrying, with hip positioning again replicating a Pavlik harness.^47^ Three further studies reported that AWSC-use improved infant physical health and development, such as strengthening musculature of the neck and spine,^52^ encouraging increased infant vocalisations^53^ and promoting development of spontaneous motor tempo.^54^

### Effects on parental mental health

Four studies examined the effect of AWSC-use on maternal^55^,^57^ and paternal^58^ mental health. One study assessed maternal comfort when babywearing to provide KMC, finding that comfort and anxiety were linked, with maternal anxiety decreasing during KMC due to close contact.^55^ In a feasibility study into the impact of AWSC-use on mental health of mothers with term babies, 29 mothers received no intervention and 32 were provided with a sling library session, free sling hire and invitation to an online AWSC community.^57^ The intervention group had significantly lower anxiety and felt more confident and closer to their babies, but no significant difference was found in the prevalence of postnatal depression. A further RCT explored how placing infants in a baby seat versus an AWSC affected the neural responses of first-time fathers.^58^ Fathers in the intervention group (n=32) showed increased amygdala reactivity to infant crying compared with the control group (n=28).

### Potential risks of using AWSC

Three topics were identified addressing potential risks and benefits of AWSC-use:

1. Risk of infant death or injury.
2. Potential risk to infant health.
3. Harm to parental health

### Risk of infant death or injury

Nine studies examined data from death and injury reports and hospital databases to identify potential risks linked with AWSC-related deaths and injuries.^59^,^67^ Sources included hospital records, US Consumer Product Safety Commission (CPSC) databases and one unverified source. The two primary risks identified which contributed to AWSC-related death or injury were asphyxiation and falls.

#### Asphyxia

Young infants, particularly those under 4 months, are at risk of positional asphyxia due to weak neck muscles and heavy heads. Difficulty controlling the head in an upright position can result in the head falling forward, forcing chin to chest,^60 63^ potentially occluding airways, restricting airflow and resulting in oxygen desaturation and asphyxia.^59 61^ Infant deaths reported by the US CPSC cite suffocation or positional asphyxia as the primary explanation for AWSC-related infant deaths.^60 62^ 19 cases of sudden deaths in AWSC reported by members of the Groupe Francophone de Reanimation et Urgences Pediatriques, found the leading mechanism of death to be suffocation; 17 infants were under 3 months, 15 infants died in autumn or winter, 18 cases occurred while caregivers were walking and in 5 cases caregivers reported carrying or positioning infants to facilitate breastfeeding.^63^ One article reported two cases with evidence of acute asphyxia, where the cause of death was cardiorespiratory arrest around 1 week after infants were found not breathing in side-lying slings.^61^

The position in which a baby is held within AWSC was identified as a potential risk, as airways can be obstructed by AWSC fabric or the caregiver’s body, or the infant’s face can be hidden from view.^60^,^63^ To reduce the risk of preventable deaths and injuries, reports recommended keeping infants’ faces visible, ensuring airways are unobstructed by fabric or caregivers’ bodies, positioning infants in an upright position and repositioning infants after feeding.^59 61 64^ Two (US) papers suggested AWSC should not be used as a routine sleep environment, recommending that sleeping infants should be removed to a clear and flat surface.^59 64^

#### Falls

Most AWSC-related injuries involved infants falling from slings and caregivers falling while carrying. Data from the Canadian Hospitals Injury Reporting and Prevention Programme (1990–1995) showed 35% of 105 reported sling-related injuries occurred due to caregiver falls/trips and 19% occurred when infants fell from AWSC. Another 19% of injuries occurred when infants were in an unworn AWSC and 6% of injuries occurred when infants were being transferred in or out of AWSC.^65^ Data from the Childhood Injury Surveillance Database in Japan found 87% of the 63 AWSC-related injury records were attributed to falls.^67^ The factors increasing the risk of injury or death via falls were how products were used; product appropriateness and design, referring to specific features of AWSC that increase fall risk, for example, wide leg openings; and product condition, such as deterioration over time resulting in failure of fabric, straps and fastenings.^65 66^

### Potential risk to infant health

Two articles examined AWSC-use in relation to specific conditions impacting infant physical health.^68 69^ As discussed, positional asphyxia is a potential risk of AWSC-use which can result in oxygen desaturation. One study investigated whether infants faced an increased risk of oxygen desaturation when carried in AWSC.^68^ Cardiorespiratory measurements, including oxygen saturation, heart rate, nasal airflow, abdominal breathing and movement, were monitored every two seconds for 20 min in 24 preterm and 12 term infants who were carried in three conditions: held vertically in a carrier with infants’ heads turned towards the parent, held horizontally in a sling in a supine position and positioned laterally in a pram. No clinically relevant changes were found in the cardiorespiratory measures recorded.^68^

In another study, researchers quantified the effects of clothing layers on thermoregulatory responses in babies under 12 months when carried in AWSC.^69^ Nine babies and mothers completed two 15-minute walking trials in a thermoneutral environment while infants wore either a sleepsuit or a vest and sleepsuit. Temperature was recorded for mothers and infants. Mothers did not perceive any changes in infant temperature, and although data indicated infant skin temperature increased by up to 1.1°C on specific body regions (eg, abdomen) the additional light layer did not significantly increase infant body temperature.

### Harm to parental health

12 studies investigated the impact of carrying on parental musculoskeletal health^70^,^78^ and cardiorespiratory systems.^79^,^81^

#### Gait and posture of carrying adult

An experimental study examined biomechanical differences in participants’ lower extremity kinematics during performance of retrieval tasks when carrying infant mannequins in three conditions: unloaded, in-arm infant carrying and AWSC-carrying.^72^ Outcomes showed individuals altered their gait mechanics to accommodate AWSC-use, however, the clinical significance of these alterations was unclear. Further studies found that other factors influence gait when infant-carrying, including footwear^71^ and type of carrier used.^70^

#### Musculoskeletal fatigue and pain in carrying adult

Six studies found AWSC-use can cause musculoskeletal fatigue and pain in carrying adults.^73^,^78^ To investigate postural differences in females holding infants during prolonged standing, participants performed 15-minute standing trials using an infant mannequin in three conditions: unloaded, in-arm carrying and carrying in AWSC.^78^ A self-report visual analogue scale for pain and discomfort in the lower body before and after each test found greater pain in at least one region of participants’ bodies in all three conditions, with more pain reported for the in-arms condition. Similar outcomes from two further studies reinforce the impact of AWSC-use on caregivers’ skeletal health.^76 77^ Prolonged AWSC-use increased the risk of lumbar lordosis and thoracic kyphosis, leading to increased pain and discomfort for caregivers^76^ and increased loading within knee joints, though not as much as in-arm carrying.^77^ These results support findings from Southeastern Nigeria where back pain was prevalent among mothers who carried their infants on their backs.^73^ No studies assessed long-term outcomes of AWSC-use for carrying adults.

The type of AWSC used can also affect caregivers’ physical health. One study investigated how the centre of pressure (COP) changes depending on the type and wearing method of AWSC. Female participants performed a 30-minute walking trial carrying a 7.6 kg infant mannequin, with three different carriers in two fit conditions: secure and loose.^75^ When carriers were worn loosely, horizontal changes to the COP were greater, with participants reporting less comfort and increased waist and thigh muscle fatigue. The x-type carrier (where straps cross the back in an ‘x’) produced the greatest muscle fatigue in shoulders, waists and thighs. The study also found that maintaining balance and ease of movement were impacted by loosely fitting carriers.^75^ An unrelated study found fatigue was influenced by infant weight, with significant differences in recorded shoulder pressure between AWSC types, and with heavier mannequins.^74^

#### Cardiopulmonary and cardiorespiratory systems in carrying adult

Three studies (all of which had a shared lead or coauthor) evaluated the effects of infant carrying methods on cardiopulmonary function, responses and perceptions of effort in women without experience of infant carrying.^79^,^81^ In one study, women walked for 6 min under four conditions: unloaded, front wrap, back wrap and hip sling using a 10 kg mannequin, with participants’ heart rate, systolic and diastolic blood pressure showing no significant differences by carrying technique, although front carrying evoked a higher cardiopulmonary response.^79^ In another study assessing female cardiopulmonary responses and perceptions of exertion to four infant-carrying methods (back, front, side and in-arms using a 6 kg mannequin), participants’ cardiorespiratory variables increased incrementally, and participants rated the in-arms method as most exerting, followed by side, front and back-carrying, respectively.^80^ A third study found lower front- and back-carrying positions produced marginally higher changes in cardiopulmonary responses compared with higher carrying positions, and participants perceived lower-back and upper-front carrying as less exerting, compared with upper-back and lower-front carrying.^81^

## Discussion

Most studies reviewed found benefits of AWSC-use for caregivers and babies. The strongest evidence in both number and robustness of studies is for AWSC-use in promoting and facilitating KMC for premature and NOWS infants, where close contact with caregivers fosters physiological stability and development. Studies found that the benefits of AWSC-use also accrue to term infants through breastfeeding, bonding and attachment, alongside physical benefits including healthy hip, neck and spine development. As AWSC-use makes infant care easier (reducing irritability and crying) and affords parents greater mobility, there are also clear benefits to parental mental health. Potential benefits with less robust evidence requiring further research include AWSC-use to support infant social development and to aid caregiving for disabled parents. Despite discussion in lay literature about the benefits of AWSC-use for infant neurological development,^82 83^ no studies directly examining this topic were identified.

This review generated evidence of AWSC-use being associated with infrequent accidental infant deaths, and physical pain and discomfort for AWSC-wearing adults, when infants are poorly positioned or AWSC are ill-fitting. AWSC-related infant death rates are low, but reports suggest they are avoidable with increased awareness around infant safety, particularly in early infancy. A UK parental survey conducted alongside this review identified three concerns around infant safety: duration of AWSC-use, sleeping in AWSC and overheating and appropriate layering.^84^ Only one reviewed study investigated thermoregulation during AWSC-use,^69^ and none examined risks or benefits of sleeping in AWSC, or the duration of AWSC-use, or whether duration of use impacted the benefits or risks discussed. Further research is needed to examine potential risks or benefits associated with AWSC in these three areas.

This review indicates that national guidance is needed to emphasise that AWSC must be well-fitted to prevent infant falls and asphyxiation. Provision of guidance to support parents in accessing well-fitting AWSC would prevent them from experiencing pain and discomfort, with further research needed on the long-term impacts of babywearing on adults’ physical health. Significantly, this review revealed no research evaluating the uptake and implementation by parents of AWSC-safety guidance, or the effectiveness of signposting parents to specialist AWSC support, a finding which informed the subsequent UK parental survey.^84^

A notable bias in the existing literature was its gendered nature—overwhelmingly, studies focused on mothers, with only one focusing on fathers. The limitations of this systematic review primarily involve the limited array of subject databases that could be searched, given available resources. Three broad-based health-related indices were chosen to maximise coverage, but we are aware of conference abstracts and specific articles in niche publications that were not captured within the scope of this review.^85^,^87^ We also excluded several studies that were not published in peer-reviewed outlets.^88 89^ Furthermore, while AWSC-use is geographically widespread, most published studies were conducted in the USA which should be borne in mind when using this review to inform UK guidance, given that AWSC-types and cause of death criteria differ between countries. Research with more diverse populations living in a greater range of settings is needed to fully understand how and why families use AWSC.

The primary outcome of this systematic review is that strong evidence exists confirming multiple benefits to babies and caregivers of AWSC-use. There is also clear evidence of accidental infant deaths and injuries occurring infrequently with AWSC-use, primarily involving falls (injuries) and asphyxiation (deaths) within a context of poor fit or positioning. Multiple studies across different countries identified that caregivers’ motivations for AWSC-use involve the desire to be responsive, to soothe babies and reduce crying and to be mobile while accomplishing this. To ensure all babies and caregivers benefit from AWSC-use without fear of accidents clear and consistent guidance must be provided to all caregivers regarding appropriate selection and safe use of AWSC. This systematic review provides the underpinning evidence that is now informing the development of UK guidance on safe AWSC-use co-produced by the research team, infant safety charities and baby-wearing educators.

## Supplementary material

10.1136/bmjpo-2026-004693 online supplemental file 1

10.1136/bmjpo-2026-004693 online supplemental file 2

## Data Availability

All data relevant to the study are included in the article or uploaded as supplementary information.
