# Supplementary material for: Safety and benefits of adult-worn slings and baby carriers: a narrative systematic review to inform guidance for parents
Source: BMJ Paediatr Open. 2026 Jun 3;10(1):e004693. doi: 10.1136/bmjpo-2026-004693 (PMC13239663; doi:10.1136/bmjpo-2026-004693)
Supplement: online supplemental file 1 [file bmjpo-10-1-s001.pdf]

Reference Number	Authors	Country Study Conducted In	Purpose / Aim	Person carrying infant	Type of Carrier & where baby carried	AWSC Risks Discussed/ Observed	Methodology/study design	Theme	Topic	Subtopic
10	Russell, N.U. (2015)	USA	to explore how non-traditional child care practices are learned and understood by mothers at free babywearing groups in Northern California.	Parents/ caregivers	Ring Slings; Wrap Slings; Soft Structured Carriers. Front; Back	Falls, Suffocation, Hip Dysplasia	Ethnographic fieldwork	Benefits, motivations, practicalities	Parental Beliefs and Perceptions	Promotion of Bonding and Attachment.
11	Little, E.E., C.H. Legare, and L.J. Carver, (2019)	USA	To test the hypothesis that maternal responsiveness to infant cues is increased by mother-infant physical contact.	Parents/ caregivers	Soft-structured carriers	None stated.	Study One: Experimental Study Two: Questionnaire Study Three: Experimental	Benefits, motivations, practicalities	Parental Beliefs and Perceptions	Promotion of Bonding and Attachment.
21	Williams, L.R. and P.R. Turner, (2020)	USA	To assess mothers experiences with babywearing and the potential of using infant carrying to improve mother-infant bonding.	Mothers	Unclear (not stated)	None stated.	Mixed methods	Benefits, motivations, practicalities	Parental Beliefs and Perceptions	Promotion of Bonding and Attachment.
22	Williams, L.R. and P.R. Turner, (2020)	USA	To assess whether infants of adolescent mothers using infant carriers have different attachment styles compared to infants of adolescent mothers who did not use infant carriers.	Mothers	Unclear (not stated)	None stated.	Intervention Study	Benefits, motivations, practicalities	Parental Beliefs and Perceptions	Promotion of Bonding and Attachment.
23	Williams, L.R (2020)	USA	To assess the impact on infant carrying on adolescent mother-infant interactions during the Still-Face Paradigm.	Mothers	Unclear (Not stated but image shows wrap sling on front)	None stated.	Intervention Study	Benefits, motivations, practicalities	Parental Beliefs and Perceptions	Promotion of Bonding and Attachment.
24	Gribble, K.D (2007)	Australia	Presents hypothesis tha the use of infant carrying helps to facilitate the close contact that children need, but provides extra support for post-instituionalised children who may not know how to be held.	Primary caregiver of post-institutionalised children	Unclear (not stated)	None stated.	Summary of scientific literature to justify hypothesis	Benefits, motivations, practicalities	Parental Beliefs and Perceptions	Promotion of Bonding and Attachment.
25	Green, K.E (2000)	USA, Canada, Iceland, Australia, Singapore	To provide demographic description of mothers who self-identify as attachment parents, and describe what these practices consist of; and to investigate the use of transitional objects in children who have higher physical contact with their mothers.	Mothers	Sling, Chest carrier, Backpack, Bucket Carrier (not stated where baby is carried)	None stated.	Descriptive Research Design (Self-report questionnare; and semi-structured telephone interviews with sample of questionnaire respondents)	Benefits, motivations, practicalities	Parental Beliefs and Perceptions	Promotion of Bonding and Attachment.
26	Grisham, L.M (2023)	USA	Synthesise evidence on biological and behaviour effects of babywearing on mothers and infants.	Mothers	Wrap Slings ; Soft Structured Carriers ; Other: Hip Sling, non-specified sling; Front; Back; Hip	None stated.	Scoping Review	Benefits, motivations, practicalities	Parental Beliefs and Perceptions	Promotion of Bonding and Attachment.
27	Douglas, P. S (2005)	Australia	Proposes hypothesis that research into gastro-oesophageal reflux disease (GORD) has been misinterpreted due to cultural assumptions. Proposes alternative interpretation of research using evolutionary biology.	Not stated	Slings; Backpacks; Front; Back	None stated.	Literature Review	Benefits, motivations, practicalities	Parental Beliefs and Perceptions	Soothing and Reduction of Crying

28	Kaley, F.M (2013)	United Kingdom	Chapter 6 (Sling Study): to investigate the effectiveness of the unique and newly-designed 'settle sling' as a method of soothing infant crying.	Mother	Soft Structured Carriers ; Other: Settle-sling (unique design); Front	None stated.	Intervention Study	Benefits, motivations, practicalities	Parental Beliefs and Perceptions	Soothing and Reduction of Crying
29	Whittle, R. (2019)	United Kingdom	To explore parental experiences of carrying children in slings and carriers.	Mother; Father	Not stated; Front; Back; Hip; Other: When speaking about data collection position is not specified. Pictures do show front, back and hip carrying. Not stated where the images come from.	None stated.	Qualitative study	Benefits, motivations, practicalities	Parental Beliefs and Perceptions	Facilitating Daily Activities
30	Wada, S., Kawate, N., Morotomi, N., Matsumiya, T., Ono, J., and Mizuma, M. (2010)	Japan	Summary of case study of 34 year old pregnant woman with Guillain-Barre Syndrome and the interventions and rehab to enable her to provide infant care post-birth.	Mother	Not stated: 'sling'; Front	None stated.	Case Study	Benefits, motivations, practicalities	Parental Beliefs and Perceptions	Enabling Infant Care for Adults with Physical Disabilities
31	Wint, A.J., Smith, D.L., and Iezzoni. L.I. (2016)	USA	To explore and describe how women with physical disabilities experience and provide infant caregiving.	Mother	Wrap Slings ; Soft Structured Carriers ; Other: Scarf, sweatshirt, belly binder; Front	None stated.	Qualitative study	Benefits, motivations, practicalities	Parental Beliefs and Perceptions	Enabling Infant Care for Adults with Physical Disabilities
32	Daniels, J.N. (2019)	United Kingdom	To discuss and explore experiences of abelism in motherhood.	Mother	Not stated.	None stated.	Authoethngraphy	Benefits, motivations, practicalities	Parental Beliefs and Perceptions	Enabling Infant Care for Adults with Physical Disabilities
34	Chavula, K., Guenther, T., Valsangkar, B., Lwesha, V., Banda, G., Wensaas, M.B., Luhanga, R., Chimtembo, L., Kinney, M.V., and Dube, Q. (2020)	Malawi	To evaluate acceptability and effectiveness of a wrap designed to facilitate Kangaroo Mother Care (KMC) in health care facilities compared to traditional chinenje wrap; and to access skin-to-skin practice in the community.	Mother	Wrap Slings ; Soft Structured Carriers ; Other: CarePlus LMC Wrap (SSC), Traditional Chitenje (Wrap); Front	None stated	Randomised Control Trial	Benefits, motivations, practicalities	Benefits of AWSC-use to infant health and development	Premature or opioid-exposed babies
35	Jegannathan, S., Natarajan, M., Solaiappan, M., Shanmugam, R., and Tilwani, S.A, (2022)	India	To improve the duration of KMC in stable, low birthweight infants over 8 weeks.	Mother	KMC sling; front	None stated.	Experimental study	Benefits, motivations, practicalities	Benefits of AWSC-use to infant health and development	Premature or opioid-exposed babies
36	Blomqvist, Y.T., Frolund, L., Rubertsson, C., and Nyqvist, K.H.(2012)	Sweden	To identify which factors parents of preterm infants perceive as supportive or barriers for KMC; to explore when and why parents dicontinue KMC	Mothers and fathers	KMC carrier; not stated	None stated.	Qualitative study.	Benefits, motivations, practicalities	Benefits of AWSC-use to infant health and development	Premature or opioid-exposed babies
37	Thapa, K., Mohan, D., Williams, E., Rai, C., Bista, S.,Mishra, S., and Hamal, P.K. (2018)	Nepal	To assess maternal perceptions of the traditional Nepalese wrap vs. CarePlus Wrap for continuation of KMC in hospital and after discharge.	Mother	Wrap Slings ; Other: Traditional Nepalese Wrap; Front	None stated.	Mixed methods feasibility study	Benefits, motivations, practicalities	Benefits of AWSC-use to infant health and development	Premature or opioid-exposed babies

38	Williams, L.R., Gebler-Wolfe, M., Grisham, L.M., and Bader, M.Y. (2020)	USA	To investigate the impact of babywearing on distress associated with NAS (neonatal abstinence syndrome - withdrawal from opioids) among infants and caregivers.	Mother ; Father ; Other: A nurse or research assistant if the immediate caregiver (Mother, Father or other family member) was not available.	Wrap Slings ; Soft Structured Carriers; front	Discussed safe positioning of infant to protect airway, and preventing infant from falling	Experimental study (linked to 37 - Williams et.al, 2020)	Benefits, motivations, practicalities	Benefits of AWSC-use to infant health and development	Premature or opioid-exposed babies
39	Williams, L.R., Grisham, L.M., Gebler-Wolfe, M., Kelsch, K., Bedrick, A., and Bader, M.Y. (2020)	USA	To examine NICU nurses perspectives of using babywearing as a method of caring for infants diagnosed with Neonatal Abstinence Syndrome (NAS)	Mother ; Father ; Other: Nurses/volunteers caring for infant	Wrap Slings ; Soft Structured Carriers: not stated but imaged wearing babies on front	None stated.	Qualitative study (linked to 36 - Williams et.al, 2020)	Benefits, motivations, practicalities	Benefits of AWSC-use to infant health and development	Premature or opioid-exposed babies
40	Rankin, L., Grisham, L.M., and Ingbar, C. (2024)	USA	To compare the effect of dynamic and static touch during babywearing amongst infants experiences Neonatal Opioid With-drawal Syndrome (NOWS).	Mother ; Father ; Other: Volunteer when parent unavailable (nurses and research assistants)	Wrap Slings ; Soft Structured Carriers; front	None stated.	Experimental study	Benefits, motivations, practicalities	Benefits of AWSC-use to infant health and development	Premature or opioid-exposed babies
41	Bhuiya, N.A., Liu, S., Muyodil, D., and Bucher, S.L (2024)	Kenya	To assess the feasibility and acceptatbility of the NeoWarm (a KMC prototype wrap); and to obtain feedback from users.	Other: Parents of premature infants and family stakeholder (grandmothers, grandfathers, adult siblings), maternal and newborn care health workers, community stakeholders and opinion leaders (e.g. traditional birth attendants, community health workers, pastors, women's group reps and village elders)	Other: NeoWarm (patented biomedical device which is a carrier and integrated elf warming swaddling pouch designed to prevent neonatal hypothermia and facilitated KMC and STS); front	None stated.	Qualitative study.	Benefits, motivations, practicalities	Benefits of AWSC-use to infant health and development	Premature or opioid-exposed babies
42	Henderson, A., and McDonagh, D. (2017)	USA	To describe the intervention of wearable incubator which enables KMC whilst providing incubator-style support.	Mother; Father	Other: wearable incubator	None stated.	Design of intervention described.	Benefits, motivations, practicalities	Benefits of AWSC-use to infant health and development	Premature or opioid-exposed babies
43	Boyce, R.M., Muhindo, E., Baguma, E., Muhindo, R., Shem, B., FranÃ§ois, R., Hawke, S., Shookâ€˜Sa, B.E., Ntaro, M., Nalusaji, A., Nyehangane, D., Reyes, R., Juliano, J.J., Siedner, M.J., Staedke, S.G., and Mulogo, E.M. (2022)	Uganda	Pilot randomised control trial seeking to assess safety, acceptability, and feasability of using permethrin-treated and untreated lesus	Mother	Lesu - traditional Ungandan wrap; back	None stated	Pilot study for Randomised Control Trial	Benefits, motivations, practicalities	Benefits of AWSC-use to infant health and development	Infection Protection

44	Boyce. R.M., Cassidy, C., Ronnie Ndizeye, R., Baguma, E., Giandomenico. D., Shook-Sa, B.E., Ntaro, M., Reyes, R., and Mulogo, E.M. (2023)	Uganda	Details study protocol for a double-blind, randomized placebo-controlled trial to assess the effectiveness of permethrin-treated lesus as a method of malaria prevention in children 6-24 months of age.	Mother	Lesu - traditional Ungandan wrap; back	None stated	Randomised Control Trial Study Protocol	Benefits, motivations, practicalities	Benefits of AWSC-use to infant health and development	Infection Protection
45	Pisacane, A., Continisio, P., Filosa, C., Tagliamonte, V., & Continisio, G. I. (2012).	Italy	To assess whether breastfeeding rates are affected by use infant carriers in the first month of life in full-term infants.	Mothers	Soft-structured carrier (not stated where carried)	None stated	Prospective Cohort Study	Benefits, motivations, practicalities	Benefits of AWSC-use to infant health and development	Breastfeeding Promotion
46	Little, E.E., C.H. Legare, and L.J. Carver (2018)	USA	To test whether mother-infant physical contact (co-sleeping or babywearing) predicts feeding in response to early hunger cues, vs. feeding using a schedule or in response to signs of distress in US mothers	Mothers	Not stated	None stated	Study One; Questionnaire Study Two: Questionnaire ad Breastfeeding Log	Benefits, motivations, practicalities	Benefits of AWSC-use to infant health and development	Breastfeeding Promotion
47	Graham, S.M., Manara, J., Chokotho, L., and Harrison, W.J. (2015)	Malawi	To present the findings of a retrospective review of the incidence of symptomatic DDH in Malawi and a systematic review of back-carrying as a potential influence on the prevalence of DDH in Malawi	Mothers	Not stated; back	Risk of DDH in infants being carried on the back	Retrospective review of DDH incidence in Malawi; and systematic review exploring positional carrying of infants and the incidence of DDH	Benefits, motivations, practicalities	Benefits of AWSC-use to infant health and development	Physical and social development
48	Fontecha, C.G., A. Coma Muñoz, and A. Catala Muñoz, (2019)	Spain	To assess infant hip positoning in different types of baby carriers using ultrasound imaging	Mothers	Soft-structured carriers; front	Hip dysplasia	Ultrasound imaging of infant hips in three different conditions (soft-structured infant carriers)	Benefits, motivations, practicalities	Benefits of AWSC-use to infant health and development	Physical and social development
49	Siddicky, S.F., Wang, J., Rabenhorst, B., Buchele, L., and Mannen, E. (2021)	USA	To assess infant hip position and leg/hip muscle activity in health infants in multiple common carrying positions (in arms vs. inward-facing carrier vs. car seat)	Not stated	Soft-structured carriers; front	Developmental hip dysplasia, Bodily injury and suffocation	Surface electromyography (EMG) electrodes were used to record muscle activity in lower extremety muscle groups in 5, 60 second position tasks, with paired sample testing used for analysis	Benefits, motivations, practicalities	Benefits of AWSC-use to infant health and development	Physical and social development
50	Vaidya, S., A. Aroojis, and R. Mehta (2021)	India	To review the existing literature regarding posnatal positioning by swaddling and babywearing as a risk factor for DDH, and to raise health professionals' awareness of this.	Not stated	Not stated	Developmental Hip displasia	Literature Review	Benefits, motivations, practicalities	Benefits of AWSC-use to infant health and development	Physical and social development

51	Siddicky, S., Eckles, J., Rabenhorst, B., and Mannen, E. (2023)	USA	To compare how hip positioning (Graf's alpha angle and femoral head coverage) varied in healthy infants and infants with mild DDH (instability) in the Pavlik harness and baby carriers.	Not stated	Soft-structured carriers; front	Developmental Hip displasia	Ultrasound imaging of infants with and without mild DDH when in a Pavlik harness, inward facing soft-structured carrier with a wide base, and inward-facing soft-structured carrier with a narrow base	Benefits, motivations, practicalities	Benefits of AWSC-use to infant health and development	Physical and social development
52	Siddicky, S.F., Bumpass, D.B., Krishnan, A., Tackett, S.A., McCarthy, R.E., and Mannen, E.M. (2020)	USA	To assess how neck and muscle activity in healthy infants differs when positioned in common positions, or in baby devices.	Not stated	Soft-structured carriers; front	None stated	Experimental study	Benefits, motivations, practicalities	Benefits of AWSC-use to infant health and development	Physical and social development
53	Mireault, G.C., B.S. Rainville, and B. Laughlin, (2018)	USA	To compare infant-parent engagment in strollers and backpack carriers	Mothers; other caregivers	Structured back-pack; back	None stated	Comparison study	Benefits, motivations, practicalities	Benefits of AWSC-use to infant health and development	Physical and social development
54	Rocha, S., Southgate, V., and Mareschal, D. (2021)	United Kingdom	To assess whether spontaneous motor temp in infantsis affected by the rate of movement experienced by infants when being carried.	Random participant	Forward-facing infant sling; front	None stated	Experimental study	Benefits, motivations, practicalities	Benefits of AWSC-use to infant health and development	Physical and social development
55	Amaliya, S., Rustina, Y. & Agustini, N. (2017)	Indonesia	To assess maternal comfort when performing KMC with three different carrier types: kangaroo pouch, thari wrap, and traditional wrap (sarung)	Mother	Wrap Slings ; Pouch Slings ; Soft Structured Carriers ; Other: Thari, Sarung (Traditional wrap); front	None stated	Experimental study	Benefits, motivations, practicalities	Effects on parental mental health	
56	Schoppmann, J., Teismann, T., Holleck-Weithmann, V.A., Hundertmark, E., Jandewerth, J., Obereiner, P., Rudolph, D., and Seehagen,S. (2023)	Germany	To investigate association between babywearing and maternal repetative negative thinking and positive mental health.	Mother	Slings; Baby carriers; not stated	None stated	Online surey about carrying behaviours and adapted German version Perseverative Thinking Questionnaire (PTQ)	Benefits, motivations, practicalities	Effects on parental mental health	
57	Wigglesworth, H., Huddy, V., Knowles, R., and Millings, A. (2023)	United Kingdom	To assess (a) feasibility of study design for a RCT (b) preliminary data about effectiveness of sling and sling support intervention	Mother	Soft Structured Carriers ; Other: Close Caboo or buckle sling. Participants had access to all types of slings at a sling library; not stated	None stated	Pilot study for randomised control trial	Benefits, motivations, practicalities	Effects on parental mental health	

58	Riem, M.M.E., Lotz, A.M., Horstman, L.I., Cima, M., Verhees, M.W.F.T., Alyousefi-van Dijk, K., van IJzendoorn, M.H., and Bakermans-Kranenburg, M.J. (2021)	The Netherlands	To investigate effects of baby carrier intervention on fathers' hormonal and neural functioning in response to infant crying.	Father	Soft-structured carrier; front	None stated	Randomised Control Trial	Benefits, motivations, practicalities	Effects on parental mental health	
59	Task Force on Sudden Infant Death, S. and R.Y. Moon (2011)	USA	To provide expanded recommendations regarding reduction of SIDS to include a safer sleep environment, including recommending that slings and carriers should not used for regular sleep.	Not stated	Not specified	Suffocation, asphyxia, entrapment	Literature review of published articles	Potential risks of using adult worn slings and baby carriers	Risk of Infant Death or Injury.	Asphyxia
60	Deppa, S.W. and E.D. Allen, (2014)	USA	To identify factors which contribute to suffocation associated with sling use and explore more effective methods of addressing these hazards.	Not stated	Ring Slings ; Wrap Slings ; Pouch Slings ; Soft Structured Carriers ; Other: 'Bag' slings and semi-structured: Not specified	suffocation in slings (positional asphyxia)	Review of literature from case files, U.S. Consumer Product Safety Commission (CPSC) injury data reports and recall files, other published reports, voluntary standards, and inspections of different types/brands of slings.	Potential risks of using adult worn slings and baby carriers	Risk of Infant Death or Injury.	Asphyxia
61	Madre, C., Rambaud, C., Avran, D., Michot, C., Sachs, P., and Dauger, S. (2014)	France	To review case study details of two infant deaths following cardiorespitory arrest whilst being carried in sling	Mother	Front; Other: Lying on side, with face towards adult body	SUDI - suffocation, asphyxia and entrapment.	Case report review.	Potential risks of using adult worn slings and baby carriers	Risk of Infant Death or Injury.	Asphyxia
62	Batra, E.K., J.D. Midgett, and R.Y. Moon, (2015)	USA	To provide analysis of mechanisms of injury and characterise risk factors associated with AWSC (and other sitting and carrying devices) for infants and young children.	Mother; Other (not specified in all cases)	Cloth; Other (not specified in all cases); Front; Other (not specified in all cases)	SIDS, Asphyxia	Retrospective review and analysis of deaths.	Potential risks of using adult worn slings and baby carriers	Risk of Infant Death or Injury.	Asphyxia
63	Bergounioux, J., Madre, C., Crucis-Armengaud, A., Briand-Huchet, E., Michard-Lenoir, A.P., Patural, H., Dauger, S., Renolleau, S., Teychéne, A.M., Henry, S. and Biarent, D., (2015.)	France, Algeria, Belgium, Luxemburg and Switzerland	To assess the circumstances in which babies die in adult-worn baby carriers	Mother	Pouch Slings ; Soft Structured Carriers	SIDS, Asphyxia, SUDI	Review of 19 case reports of infant death linked to AWSC use.	Potential risks of using adult worn slings and baby carriers	Risk of Infant Death or Injury.	Asphyxia

64	Rholdon, R., (2017)	USA	To demonstrate the need for all parents, hospital staff, and caregivers to understand the potential risks associated with using sitting devices (including AWSC) for routine sleep.	Caregivers	Not specified	SIDS, SUDI, suffocation, strangulation	Literature Review	Potential risks of using adult worn slings and baby carriers	Risk of Infant Death or Injury.	Asphyxia
65	Brooks, J. and F. Finlay (2000)	UK and Canada	Summary of case study in which baby fell from sling, and discussion of existing literature	Mother	Not specified	Falling from sling	Case report and literature review	Potential risks of using adult worn slings and baby carriers	Risk of Infant Death or Injury.	Falls
66	Frisbee, S.J. and H. Hennes, (2000)	USA	To examine and describe injuries experienced by infants associated with adult-worn child carriers.	Not stated	Not specified, front; back; other unspecified	Product recalls due to structural issues, infant falls, and caregiver falls whilst carrying.	Literature search from injury databases, and product safety databases.	Potential risks of using adult worn slings and baby carriers	Risk of Infant Death or Injury.	Falls
67	Zhang, K., Nishidab, Y., Kitamurab, K., and Mikamia, Y. (2019)	Japan	To use an injury description framework, to describe the role of each object involved in the process that resulted in injury to the child.	Not stated	Baby sling; buckle carriers	Falls, pinching	Matrix analysis	Potential risks of using adult worn slings and baby carriers	Risk of Infant Death or Injury.	Falls
68	Stening, W., Nitsch, P., Wassmer, G., and Roth, B. (2002)	Germany	To assess whether the use of infant slings is associated with clinically relevant changes to cardo-respiratory measurements.	Mother; Father	Not specified; 'sling'; Front	Changes to cardiorespiratory measurements	Experimental Study	Potential risks of using adult worn slings and baby carriers	Potential Risk to infant health	Potential Risk to infant health
69	Filingeri, D., Cowley, H., Merrick, C., Parenting Science Gang and Filingeri, V.L. (2020)	USA	To quanitfy the effect of baby clothing layers when babywearing on thermoregulatory responses in infants under 1 year.	Not stated	wrap sling; front	Overheating due to overwrapping/excessive clothing, leading to increased risk of death	Experimental study	Potential risks of using adult worn slings and baby carriers	Potential Risk to Infant Health	Potential Risk to Infant Health
70	Brown, M.B., C.J. Digby-Bowl, and S.D. Todd, (2018)	United Kingdom	To determinte changes in ground reaction forces experienced when carrying an infant on the chest; and to determine wheher carrier structure affects the foot-ground interaction experienced by the caregiver.	Random participants (male and female)	Soft-structured carriers; Front	Referenced TICKS and need to carry infant high on chest and close enough to kiss when considering positioning of baby on carrying adult.	Experimental study	Potential risks of using adult worn slings and baby carriers	Harm to Parental Health	Gait and posture of carrying adult
71	Hyun, S., and Ryew, C., (2018)	Korea	To investigate the effect of infant carrying on leg mechanics when wearing high heeled shoes.	Mother	Soft-structured carrier; back	Potential risk of injury/falls to person wearing heels and carrying infant.	Experimental study.	Potential risks of using adult worn slings and baby carriers	Harm to Parental Health	Gait and posture of carrying adult
72	Havens, K.L., Severin, A.C., Bumpass, D.B., and Mannen, E.M. (2020)	USA	To determine the biomechanical impact of different carrying methods during a gait and retrieval task.	Random participants (nulliparous women)	Soft-structured carrier; front	None to infant	Experimental study.	Potential risks of using adult worn slings and baby carriers	Harm to Parental Health	Gait and posture of carrying adult

73	Ojukwu, C.P., Anyanwu, G.E., Anekwu, E.M., Chukwu, S.C., and Fab-Agbo, C. (2017	Nigeria	To investgiate the most commonly used infant carrying methods, including reasons for carrying, associated maternal muskoskeletal disorders, and sociodemographic status.	Mothers	Not stated; Front, Back, Other	Muscoskeletal injuries to mother due to babycarrying	Questionnaire	Potential risks of using adult worn slings and baby carriers	Harm to Parental Health	Musculoskeletal fatigue and pain in carrying adult
74	Wu, C.Y., H.R. Huang, and M.J. Wang, (2017)	Taiwan	To investigate the physiological response of the carrying adult when using front baby carriers, and see if they differed between genders, baby carrier types, and baby weights.	Random participants (male and female)	Ring Slings ; Soft Structured Carriers; Front	Suffocation in infants	Experimental study (Nested factorial design)	Potential risks of using adult worn slings and baby carriers	Harm to Parental Health	Musculoskeletal fatigue and pain in carrying adult
75	Lee, H. and K.H. Hong, (2018)	South Korea	To explore how the centre of pressure (COP) changes dependent on type and wearing method of baby carrier.	Mothers	Soft Structured Carriers ; X-type, H-Type and H-Hip Type; Front	Hip Dysplasia	Experimental study	Potential risks of using adult worn slings and baby carriers	Harm to Parental Health	Musculoskeletal fatigue and pain in carrying adult
76	Schmid, S., Stauffera, M., JÃ¤ger, J., List, R., and Lorenzettib, S. (2019)	Switzerland	To assess immediate effects of different infant-sling carrying techniques on neuromechanics of the spine	Random participants (female)	Ring Slings; front; hip	None stated	Experimental study	Potential risks of using adult worn slings and baby carriers	Harm to Parental Health	Musculoskeletal fatigue and pain in carrying adult
77	Williams, L., T. Standifird, and M. Madsen (2019)	USA	To explore how babywearing, carrying infant in-arms, and walking unloaded afect lower extermity joint movement during prolonged walking, and how these compare to one another.	Random participants (female)	Soft Structured Carriers; Front	None stated	Experimental study	Potential risks of using adult worn slings and baby carriers	Harm to Parental Health	Musculoskeletal fatigue and pain in carrying adult
78	Mannen, E.M., Havens, K.L., Kahney, A., and Nelson-Wong, E. (2020)	USA	To quantify the postural differences between women holding infants in arms; in baby carriers; and in an unloaded condition during prolonged standing periods.	Random participants (nulliparous women)	Soft Structured Carriers; Front	Muscoskeletal injuries to mother due to babycarrying	Experimental study	Potential risks of using adult worn slings and baby carriers	Harm to Parental Health	Musculoskeletal fatigue and pain in carrying adult
79	Ojukwu, C.P., Nnamoko, C.I., Okemuo, A.J., Ede, S.S., Ilo, I.J., Ikele, C.N., and Akinola, T.O. (2020)	Nigeria	To investigate how the position of the infant on the front/back of the carrying woman affects cardiopulmonary responses during simulated back and front infant carrying tasks.	Random participants (nulliparous women)	Front baby carrier and wrap (unclear if different types of carrier); front; back	Impact of position of infant on front/back of mother on maternal physiological chance	Experimental study	Potential risks of using adult worn slings and baby carriers	Harm to Parental Health	Cardiopulmonary and cardiorespiratory systems in carrying adult
80	Mbada, C.E., Adebayo, O.S., Olaogun, M.O., Johnson, O.E., Ogundele, A.O., Ojukwu, C.P., Akinwande, O.A., and Makinde, M.O. (2022)	Nigeria	To evaluate the effect of three different types of infant carrying techniques on cardiopulmonary function, metabolic expenditure, fatigue demand and locomotion.	Random participants (females)	Soft-structured carriers; front. Back; hip	None stated	Experimental study	Potential risks of using adult worn slings and baby carriers	Harm to Parental Health	Cardiopulmonary and cardiorespiratory systems in carrying adult
81	Ojukwu, C.P., Okafor, C.J., Chukwu, S.C., Anekwu, E.M., and Okemuo, A.J. (2019)	Nigeria	To investigate the cardiopulmonary and perceptual responses to four Infant Carrying Methods (ICM) (back, front, side and in-arms)	Random participants (females)	Wrap Slings ; Other: Front carrier and wrap carrier unclear; front; back; hip	None stated	Experimental study	Potential risks of using adult worn slings and baby carriers	Harm to Parental Health	Cardiopulmonary and cardiorespiratory systems in carrying adult
