# Supplementary material for: Safety and benefits of adult-worn slings and baby carriers: a narrative systematic review to inform guidance for parents
Source: BMJ Paediatr Open. 2026 Jun 3;10(1):e004693. doi: 10.1136/bmjpo-2026-004693 (PMC13239663; doi:10.1136/bmjpo-2026-004693)
Supplement: online supplemental file 2 [file bmjpo-10-1-s002.docx]

**Data Extraction Template**

**General Information**

- Study ID
- Title
- Publication Year
- Authors
- Type of Report
- Country in which Study was conducted
- Study Population or Participants

**Study Characteristics**

Key Topics

- Risks or Benefits of using slings, wraps or carriers in baby wearing practices
- Parent motivation or perceptions of using slings, wraps or carriers in infant caregiving.
- Relationship of use of slings/wraps/carriers to infant health and/or SUDI
- Relationship of use of slings/wraps/carriers to parental physical and mental health
- Sling, Wrap or Carrier Practices within different countries and/or cultures

**Specific Sling Information**

- Types of Carriers investigated (Ring Slings; Wrap Slings; Pouch Slings; Soft Structured Carriers; Other)
- Who is carrying the baby? (Mother; Father; Other)
- Where is baby carried? (Front; Back; Hip; Other)

**Study Summary**

- Aim of study
- What known risks are observed/discussed in the study?
- Methodology
- Key Results
- Critical Analysis
- Conclusions or Future Research Noted
- Additional Notes
