# Supplementary material for: Safety and benefits of adult-worn slings and baby carriers: a narrative systematic review to inform guidance for parents
Source: BMJ Paediatr Open. 2026 Jun 3;10(1):e004693. doi: 10.1136/bmjpo-2026-004693 (PMC13239663; doi:10.1136/bmjpo-2026-004693)
Supplement: Supplementary file 3 [file bmjpo-2026-004693.reviewer_comments.pdf]

**PEER REVIEW HISTORY**

BMJ Paediatrics Open publishes all reviews undertaken for accepted manuscripts. Reviewers are asked to complete a checklist review form and are provided with free text boxes to elaborate on their assessment. These free text comments are reproduced below.

**ARTICLE DETAILS**

| | |
|---|---|
| **TITLE (PROVISIONAL)** | Safety and benefits of adult-worn slings and baby carriers: a narrative systematic review to inform guidance for parents |
| **AUTHORS** | Lovell-Kennedy, Sophie Rebecca<br>Strong, Rebecca L.J.<br>Grieve, Laura M<br>Ball, Helen Louise |

**VERSION 1 – REVIEW**

| | |
|---|---|
| **REVIEWER NAME** | Thomas, Victoria |
| **REVIEWER AFFILIATION** | Newcastle Upon Tyne Hospitals NHS Foundation Trust, Great North Children's Hospital |
| **REVIEWER CONFLICT OF INTEREST** | I have previously been a voluntary unpaid member of the Lullaby Trust Scientific Committee. |
| **AI DISCLOSURE** | No, I have not used AI |
| **DATE REVIEW RETURNED** | 16-Mar-2026 |

| | |
|---|---|
| **GENERAL COMMENTS** | This is a comprehensive and timely review of a significant number of studies which navigates the reader expertly through the literature whilst acknowledging the gaps and omissions. It is useful to address the questions of families and professionals.<br><br>There is a typo on the data extraction template ('kay' instead of key) and the first page of the summary table of included studies appears to be missing.<br><br>I have no other alterations to suggest and recommend this article for publication. |

| | |
|---|---|
| **REVIEWER NAME** | Chiu, Serena |
| **REVIEWER AFFILIATION** | The University of Sydney |
| **REVIEWER CONFLICT OF INTEREST** | No Conflict of Interest |
| **AI DISCLOSURE** | No, I have not used AI |
| **DATE REVIEW RETURNED** | 25-Mar-2026 |

| | |
|---|---|
| **GENERAL COMMENTS** | 1. Typo? "Outcomes the from extracted data clustered in two main themes"<br>2. Might it be helpful to provide an example for the "indirect evidence" for babies' biological need for close contact, just as you have done when explaining how AWSC-use can promote secure attachment etc.<br>3. "Most studies were conducted in the USA". Since the work aims to lay groundwork for guidelines for UK, the authors may benefit from including a comment addressing this limitation (i.e., why these |

| | |
|---|---|
| | findings may or may not be generalisable to a UK setting).<br>4. In addition with the above point, the authors may find it beneficial to summarise in the Discussion how the work contributes towards recommendations for UK national guidance.<br>5. "Infection Protection" listed as a benefit of AWSC-use. The study described in this section seems to indicate that the effectiveness of AWSC-use has yet to be explored ("A subsequent RCT protocol proposed to examine the effect of these wraps on clinical malaria incidence"). Is there a way to make it clearer that this may be a potential benefit of AWSC-use, perhaps by modifying the subtheme name? |

**VERSION 1 – AUTHOR RESPONSE**

Reviewer: 1
Victoria Thomas, Newcastle Upon Tyne Hospitals NHS Foundation Trust
**Comments to the Author**
This is a comprehensive and timely review of a significant number of studies which navigates the reader expertly through the literature whilst acknowledging the gaps and omissions. It is useful to address the questions of families and professionals.

Thank you for these positive comments.

There is a typo on the data extraction template ('kay' instead of key) and the first page of the summary table of included studies appears to be missing.

Thank you for spotting the typo, this has been fixed.

The first page of the summary Table is not missing, but we understand why it might appear to be missing as the numbering begins at 10. This is because we numbered all references consecutively and the previous 9 references were cited in the background section.

I have no other alterations to suggest and recommend this article for publication.

Many thanks

Reviewer: 2
Ms. Serena Chiu, The University of Sydney
**Comments to the Author**
1. Typo? "Outcomes the from extracted data clustered in two main themes"

Thank you for spotting the typo, this has been fixed.

2. Might it be helpful to provide an example for the "indirect evidence" for babies' biological need for close contact, just as you have done when explaining how AWSC-use can promote secure attachment etc.

We have revised this sentence to state; Babies' biological need for close contact was noted in some papers via indirect evidence such as how AWSC-use helped babies thrive, cry less, and breastfeed more[10,25].
3. "Most studies were conducted in the USA". Since the work aims to lay groundwork for guidelines for UK, the authors may benefit from including a comment addressing this limitation (i.e., why these findings may or may not be generalisable to a UK setting).

Thank you for this suggestion. We have added to the original statement: …. which should be borne in mind when using this review to inform UK guidance given that AWSC-types and cause of death criteria differ between countries.

4. In addition with the above point, the authors may find it beneficial to summarise in the Discussion how the work contributes towards recommendations for UK national guidance.

Thanks for this suggestion. We have added the comment that This systematic review provides the underpinning evidence that is now informing the development of UK guidance on safe AWSC-use co-produced by the research team, infant safety charities and baby-wearing educators.
We cannot currently add details about the new guidance as its release is embargoed until mid-May.

5. "Infection Protection" listed as a benefit of AWSC-use. The study described in this section seems to indicate that the effectiveness of AWSC-use has yet to be explored ("A subsequent RCT protocol proposed to examine the effect of these wraps on clinical malaria incidence"). Is there a way to make it clearer that this may be a potential benefit of AWSC-use, perhaps by modifying the subtheme name?

Thank you for picking up on this. We have modified the title to say *Potential Infection Protection*

## VERSION 2 – REVIEW

| | |
|---|---|
| **REVIEWER NAME** | Thomas, Victoria |
| **REVIEWER AFFILIATION** | Newcastle Upon Tyne Hospitals NHS Foundation Trust, Great North Children's Hospital |
| **REVIEWER CONFLICT OF INTEREST** | No Conflict of Interest |
| **AI DISCLOSURE** | No, I have not used AI |
| **DATE REVIEW RETURNED** | 19-Apr-2026 |

| | |
|---|---|
| **GENERAL COMMENTS** | Thank you for addressing previous concerns |

| | |
|---|---|
| **REVIEWER NAME** | Chiu, Serena |
| **REVIEWER AFFILIATION** | The University of Sydney |
| **REVIEWER CONFLICT OF INTEREST** | No Conflict of Interest |
| **AI DISCLOSURE** | No, I have not used AI |
| **DATE REVIEW RETURNED** | 27-Apr-2026 |

| | |
|---|---|
| **GENERAL COMMENTS** | N/A |
